# Aberrant Membrane Structures in Hypervesiculating *Escherichia coli* Strain *ΔmlaEΔnlpI* Visualized by Electron Microscopy

**DOI:** 10.3389/fmicb.2021.706525

**Published:** 2021-08-11

**Authors:** Yoshihiro Ojima, Tomomi Sawabe, Mao Nakagawa, Yuhei O. Tahara, Makoto Miyata, Masayuki Azuma

**Affiliations:** ^1^Department of Applied Chemistry and Bioengineering, Graduate School of Engineering, Osaka City University, Osaka, Japan; ^2^Graduate School of Science, Osaka City University, Osaka, Japan; ^3^The OCU Advanced Research Institute for Natural Science and Technology (OCARINA), Osaka City University, Osaka, Japan

**Keywords:** *Escherichia coli*, outer membrane vesicle, quick-freeze deep-etch electron microscopy, plasmolysis, multilamellar outer membrane vesicle

## Abstract

*Escherichia coli* produces extracellular vesicles called outer membrane vesicles (OMVs) by releasing a part of its outer membrane. We previously reported that the combined deletion of *nlpI* and *mlaE*, related to envelope structure and phospholipid accumulation in the outer leaflet of the outer membrane, respectively, resulted in the synergistic increase of OMV production. In this study, the analysis of Δ*mlaE*Δ*nlpI* cells using quick-freeze, deep-etch electron microscopy (QFDE-EM) revealed that plasmolysis occurred at the tip of the long axis in cells and that OMVs formed from this tip. Plasmolysis was also observed in the single-gene knockout mutants Δ*nlpI* and Δ*mlaE*. This study has demonstrated that plasmolysis was induced in the hypervesiculating mutant *E. coli* cells. Furthermore, intracellular vesicles and multilamellar OMV were observed in the Δ*mlaE*Δ*nlpI* cells. Meanwhile, the secretion of recombinant green fluorescent protein (GFP) expressed in the cytosol of the Δ*mlaE*Δ*nlpI* cells was more than 100 times higher than that of WT and Δ*nlpI*, and about 50 times higher than that of Δ*mlaE* in the OMV fraction, suggesting that cytosolic components were incorporated into outer-inner membrane vesicles (OIMVs) and released into the extracellular space. Additionally, QFDE-EM analysis revealed that Δ*mlaE*Δ*nlpI* sacculi contained many holes noticeably larger than the mean radius of the peptidoglycan (PG) pores in wild-type (WT) *E. coli*. These results suggest that in Δ*mlaE*Δ*nlpI* cells, cytoplasmic membrane materials protrude into the periplasmic space through the peptidoglycan holes and are released as OIMVs.

## Introduction

Outer membrane vesicles (OMVs) are nanosized, spherical, bilayered membranous structures with a diameter of 20–250 nm. OMVs are normally discharged from the surface of Gram-negative bacteria, including *Escherichia coli* (Schwechheimer and Kuehn, 2015; Toyofuku et al., 2019). OMVs contain outer membrane proteins, lipids, periplasmic proteins, lipopolysaccharides, RNA, and DNA.

Vesicle formation is promoted by a disturbance in growth, turnover in cell wall components, or exposure to antibiotics (Schwechheimer and Kuehn, 2015; Toyofuku et al., 2019). Particularly, envelope stress and phospholipid accumulation in the outer membrane have been established as the dominant factors of OMV production in Gram-negative bacteria (Toyofuku et al., 2019). The cell envelope, comprising a peptidoglycan (PG)-containing cell wall and a lipopolysaccharide-containing outer membrane, is organized in wild-type (WT) Gram-negative bacteria. In *E. coli*, the *nlpI* gene encodes an outer membrane lipoprotein (Lpp; Ohara et al., 1999). The deletion of *nlpI* gene decreases the crosslinks lipoprotein–peptidoglycan (Lpp-PG): the amount of Lpp-PG crosslinks is approximately 40% lower in the hypervesiculating *nlpI* mutant than in the WT strain and caused changes in envelope structure (McBroom and Kuehn, 2007; Schwechheimer et al., 2015). The balance between the breakdown and synthesis of PG is modified in the *nlpI* mutant, preventing the formation of proper crosslinks indirectly leading to OMV production (Schwechheimer and Kuehn, 2015). Other studies showed phospholipid accumulation in the outer leaflet of the outer membrane causes the dysfunction associated with disruption of VacJ (also known as MlaA) and Yrb, which encodes an ATP-binding cassette transport system, thus increasing OMV production in Gram-negative bacteria *Haemophilus influenzae* and *Vibrio cholerae* (Roier et al., 2016). This observation confirms a genome-wide assessment of OMV production in *E. coli* that demonstrated the increase in vesiculation in the *mlaA* (*vacJ*) and *mlaE* (*yrbE*) mutants (Kulp et al., 2015). Thus, both changes in envelope structure and phospholipid accumulation are essential endogenous factors promoting OMV production. Even more interesting, in our previous study, the double-gene knockout mutant Δ*mlaE*Δ*nlpI* of *E. coli* that has both properties of changes in envelope structure and phospholipid accumulation demonstrated the synergistic increase of OMV production (Ojima et al., 2020). Furthermore, green fluorescent protein (GFP) fused with the outer membrane protein W (OmpW) was expressed in OMVs to evaluate their capacity for protein secretion. Western blot analysis showed that OmpW-GFP secretion by the OMVs in the culture medium of Δ*mlaE*Δ*nlpI* strain reached 3.3 mg/L; 500 times that of the WT strain (Ojima et al., 2020). Since the OMV production by Δ*mlaE*Δ*nlpI* was 30 times that of the WT strain, a 500-fold increase in OmpW-GFP secretion by Δ*mlaE*Δ*nlpI* could not be fully explained by an increase in OMV production.

Several types of novel OMV, such as outer-inner membrane vesicles (OIMVs) or explosive outer membrane vesicles (EOMVs), have been reported (Toyofuku et al., 2019). The first evidence for OIMVs was provided *via* a transmission electron cryomicroscopy study of the supernatant of *Shewanella vesiculosa* M7 that clearly demonstrated the production of double bilayered OMVs (Pérez-Cruz et al., 2013). Moreover, this study showed that the OIMVs contained intracellular molecules such as DNA. Conversely, the Δ*tolB* mutant of *Buttiauxella agrestis* produced multilamellar and multivesicular OMVs (M-OMVs). M-OMV is defined to contain triple-lamellar membrane vesicles and multiple vesicle-incorporating vesicles. The deletion of *tolB*, which encodes part of the Tol-Pal system, causes the production of multiple types of vesicles and increases the overall vesicle production in JCM 1090 T, a hypervesiculating strain of *B. agrestis* (Takaki et al., 2020). The visualization of the intracellular compartments of the Δ*tolB* cells by quick-freeze deep-etch electron microscopy (QFDE-EM) showed that vesicles had accumulated in the broad periplasm, likely because of the dissociation of the outer membrane from the underlying PG. The outer membrane was invaginating to create vesicles, and the precursor of M-OMVs was present in the cell. Considering these reports together with our previous results, the double-deletion strain of *E. coli* Δ*mlaE*Δ*nlpI* may produce M-OMVs, including OIMVs. Such a phenomenon may explain the 500-fold increase in GFP secretion that cannot be explained only by increased OMV production.

In this study, we investigated the mechanism underlying the OMV hypervesiculation in the single-deletion mutants Δ*mlaE* and Δ*nlpI* and the double-deletion mutant Δ*mlaE*Δ*nlpI*, using QFDE-EM. We also examined the properties of the OMVs produced by a double-deletion mutant. Finally, we discussed the possibility of OIMV production in *E. coli*.

## Materials and Methods

### Bacterial Strains and Culture Conditions

The strains and plasmids used in this study were listed (Table 1). The WT *E. coli* K-12 strain BW25113 and its derivatives were obtained from the National BioResource Project [National Institute of Genetics (NIG), Mishima, Japan; Baba et al., 2006]. The double-gene knockout mutants were constructed by P1 transduction using the P1kc phage (Ojima et al., 2020). Each strain was transformed with pCA24N-*gfp* (Ojima et al., 2018) to express the His-tagged GFP in the cytosol.

**Table 1:** *Escherichia coli* strains and plasmid used in this study.

Strains	Note	Reference
*E. coli*		
BW25113	*rrnB*T14 *ΔlacZ*WJ16 *hsdR*514 *ΔaraBAD*AH33	Baba et al., 2006
*ΔnlpI* (JW3132)	BW25113, *ΔnlpI*::*FRT-Km-FRT*	Baba et al., 2006
*ΔmlaE* (JW3161)	BW25113, *ΔmlaE*::*FRT-Km-FRT*	Baba et al., 2006
*ΔmlaE* *ΔnlpI*	*ΔmlaEΔnlpI*::*FRT-Km-FRT*	Ojima et al., 2020
Plasmid		
pCA24N-*gfp*	pCA24N carrying *gfp* under P_T5-lac_ control, Cm^r^	Ojima et al., 2018

The *E. coli* cells were cultured in lysogeny broth (LB; 10 g/L Bacto™ Tryptone, 5 g/L yeast extract, and 10 g/L NaCl). The culture medium for the strains harboring pCA24N-*gfp* was supplemented with 50 mg/L chloramphenicol and 1 mM IPTG. All the test cultures were precultured in LB for 18 h at 37°C and inoculated into 100 ml of fresh LB in a 500 ml baffled conical flask to achieve the optical density at 660 nm (OD_660_) of 0.01. The cultures were incubated on an NR-30 rotary shaker (Taitec, Osaka, Japan) at 140 strokes/min. Cell growth was measured at OD_660_.

### Cell Volume Distribution Analysis Using qNano System *via* Scanning Ion Occlusion Sensing

Cells of each strain were harvested at 3 and 24 h after inoculation by centrifugation at 10,000 × *g* and 4°C for 10 min. Cells were washed by filtrated PBS (pH7.5) and resuspended in PBS. Cell size distribution analysis was conducted using the qNano system (qNano, IZON Science Ltd., Christchurch, New Zealand) equipped with Nanopore NP-1000. Scanning ion occlusion sensing allows single-particle measurements since colloids or biomolecular analytes or both are driven through pores one at a time. Particles crossing the nanopore are detected as a transient change in the ionic current flow, also denoted as a blockade event whose amplitude is the blockade magnitude. As blockade magnitude is proportional to particle size, accurate particle sizing can be achieved after calibration with a known standard. Here, size calibration was conducted using CPC1000 standard particles.

### Quick-Freeze, Deep-Etch EM

For QFDE-EM analysis, bacterial cells were first washed with PBS (pH 7.5) twice, resuspended in HEPES-NaCl buffer or 15% (v/v) glycerol solution, and centrifuged (Tulum et al., 2019; Takaki et al., 2020). Then, a rabbit lung slab, mica flakes, and bacterial cell pellets were placed in this order onto a paper disk attached to an aluminum disk, and the samples were quickly frozen using liquid helium with a CryoPress (Valiant Instruments, St. Louis, MO, United States). The rabbit lung slab and the mica flakes function as shock absorber in quick freezing and flat background in observation, respectively. The specimens were stored in a chamber at −180°C using a JFDV freeze-etching device (JEOL, Tokyo, Japan). After the samples’ temperature was increased to −120°C, they were freeze-fractured with a knife and freeze-etched at −104°C for 15 min. The freeze-etched step was omitted when 15% (v/v) glycerol was used as the solvent. Subsequently, the samples were coated with platinum at a thickness of 2 nm and a rotary shadowing angle of 20° and then coated with carbon at a rotary shadowing angle of 80°. Next, the replicas were floated on full-strength hydrofluoric acid, rinsed in water, cleansed with commercial bleach containing sodium hypochlorite, rinsed in water, and finally placed onto 400-mesh Cu grids. The replica specimens were observed with transmission electron microscopy (TEM) using a JEM-1010 (JEOL).

### Isolation and Observation of Outer Membrane Vesicles

Outer membrane vesicles were isolated as previously described (Gujrati et al., 2014) with some modifications. After incubation for 24 h, 100 ml of the *E*. *coli* culture was centrifuged at 10,000 × *g* for 10 min at 4°C to remove the cells. Then, the supernatant was passed through a 0.45 μm filter. Ammonium sulfate was added at the final concentration of 400 g/L to incubate for 1 h at room temperature to precipitate the contents. Crude OMVs were obtained *via* centrifugation at 11,000 × *g* for 30 min at 4°C. The crude extracts were dissolved in 500 μl of 15% (v/v) glycerol and concentrated using a CS100FNX ultracentrifuge (Hitachi Koki Co., Tokyo, Japan) at 109,000 × *g* for 1 h. The OMV pellets were resuspended in 100 μl of 15% (v/v) glycerol. The resulting OMV samples were 1,000 times more concentrated than that in the original culture because of the volume decreasing from 100 ml to 100 μl. The OMV samples were placed onto a 200-mesh copper grid and negatively stained with 4% uranyl acetate for TEM observation under a JEM-2100 (JEOL).

### Evaluation of OMV Production and GFP Secretion Through OMVs

Ten microliters of the isolated OMVs or a culture of *E. coli* cells was analyzed *via* sodium dodecyl sulfate–polyacrylamide gel electrophoresis (SDS-PAGE) and Coomassie Blue staining. OMV production was quantified as previously described (Schwechheimer and Kuehn, 2013) with some modifications. The OMV concentration was measured by quantifying the band at approximately 37 kDa in the SDS-PAGE gel using the Image J software (National Institutes of Health, Bethesda, MD, United States). The OMV production of each mutant was shown as relative value to WT.

Outer membrane vesicles were also quantified with a lipophilic dye FM4-64 according to a previously described method with minor modifications (McBroom et al., 2006; Manning and Kuehn, 2011; Klimentová and Stulík, 2015). Isolated OMVs were incubated with 5 μg/ml FM4-64 (Molecular Probes/Thermo Fisher, Waltham, MA, United States) in PBS (pH 7.5) for 20 min. Then, OMVs were measured at the excitation and emission wavelengths of 558 and 734 nm, respectively, using an INFINITE 200 PRO spectrofluorophotometer (TECAN, Switzerland). The OMV sample without staining by a lipophilic dye (FM4-64) or the lipophilic dye alone was used as the negative controls.

The His-tagged GFP included in OMV samples was analyzed *via* western blotting by first transferring the protein from a gel to an Immobilon-P membrane sheet (Merck Millipore, Billerica, MA, United States) using the semidry transfer method. The blot was hybridized with an anti-histidine-tag primary monoclonal antibody (Medical & Biological Laboratories Co., Nagoya, Japan) and then a secondary antibody, the anti-mouse immunoglobulin G (whole molecule)–alkaline phosphatase (Sigma-Aldrich, St. Louis, MO, United States). The hybridization signals were detected using a BCIP-NBT Solution Kit for Alkaline Phosphatase Stain (Nacalai Tesque, Kyoto, Japan). The level of the target protein was quantified by analyzing the bands on the western blot band using densitometry. GFP secretion was quantified on the basis of the intensity of a GFP standard sample (50 μg/L) with a His-tag (Sino Biological Inc., Beijing, China). The GFP produced in each *E. coli* cell was also analyzed *via* western blotting. In the case of cell analysis, a 10 μl sample at OD_660_ = 0.3 was loaded into each well of gel to include the same amount of cells.

The GFP-derived fluorescence of the OMV samples was observed at an excitation wavelength of 488 nm using a confocal laser scanning microscope (CLSM; DM6000B, Leica, Wetzlar, Germany) with the TCS SP8 software.

### Determination of OMV Size Using Dynamic Light Scattering

Outer membrane vesicles isolated by ultracentrifugation as described above were resuspended in PBS buffer (pH 7.5). After dilution of OMV samples with pure water, dynamic light scattering (DLS) measurements were conducted at 25°C using a ZetaSizer NanoSeries equipped with a HeNe laser source (*λ* = 633 nm; Malvern Instruments Ltd., Worcestershire, United Kingdom) and analyzed using the Dispersion Technology Software (Malvern Instruments Ltd.). For each sample, the autocorrelation function was the average of five 10 s runs and then repeated approximately 3–6 times. CONTIN analysis was subsequently used for the number vs. hydrodynamic size profiles to study the dispersions.

### Preparation of *E. coli* Sacculi and Observation *via* TEM or QFDE-EM

After 24 h in culture, the *E. coli* cells were collected *via* centrifugation at 10,000 × *g* and 4°C for 10 min and suspended in PBS buffer (pH 7.5). These steps were repeated once. Then, the cells were resuspended in 10% SDS (w/v) and incubated at 95°C for 12 h. The sacculi were harvested *via* centrifugation at 200,000 × *g* and 20°C for 40 min, washed three times in Milli-Q water, and observed using TEM with negative staining or QFDE-EM.

## Results

### Properties of Cells and OMVs in Each *E. coli* Mutant

The cell growth, cell volume, relative OMV production, and average OMV size of each strain of hypervesiculating *E. coli*, including the single-deletion mutants Δ*mlaE* and Δ*nlpI* and the double-deletion mutant Δ*mlaE*Δ*nlpI* at the end of a 24 h culture, were measured (Table 2). The OD_660_ and relative OMV production based on the SDS-PAGE analysis were taken from our previous study (Ojima et al., 2020).

**Table 2:** Summary of cell growth, cell size, relative OMV production, and OMV size of each *E. coli* strain.

	Culture time (h)	WT	*ΔnlpI*	*ΔmlaE*	*ΔmlaE* *ΔnlpI*	Reference
OD_660_ [−]	24	5.1 ± 0.6	4.1 ± 0.7	5.2 ± 0.3	2.9 ± 0.2^*^	Ojima et al., 2020
Relative OMV production (SDS-PAGE) [−]	24	1	7.0 ± 1.2^*^	5.3 ± 1.5^*^	29.4 ± 5.6^*^	Ojima et al., 2020
Relative OMV production (FM4-64) [−]	24	1	5.8 ± 1.0^*^	8.7 ± 0.7^*^	13.8 ± 3.9^*^	This study
Average OMV size [nm]	24	83.9 ± 0.3	81.7 ± 0.3	65.9 ± 0.6^*^	100.4 ± 0.5^*^	This study
Cell volume [fL]	3	1.08 ± 0.27	0.85 ± 0.26^*^	1.04 ± 0.27	1.08 ± 0.28	This study
	24	0.52 ± 0.18	0.39 ± 0.15^*^	0.41 ± 0.14^*^	0.39 ± 0.20^*^	This study

The data were obtained from three independent experiments (*n* = 3). The values of average OMV size are the means of average values from three independent experiments. The dimensionless quantity was indicated by the symbol [−]. The statistical differences were found between WT values and the values marked with asterisks determined by the Student’s *t*-test (*p* < 0.05).

In this study, OMV production was quantified with a lipophilic dye, FM4-64. The relative OMV production by Δ*nlpI* and Δ*mlaE* strains assayed with FM4-64 was 5.8 and 8.7 times, respectively, that of the WT strain (Table 2), consistent with the results from the SDS-PAGE analysis in our previous study. Meanwhile, the OMV production by Δ*mlaE*Δ*nlpI* strain was determined to be approximately 14 times higher than that of the WT strain, in contrast to the 30-fold difference revealed *via* the SDS-PAGE in the previous study. Although there was a deviation depending on the measurement method, it was again confirmed that the OMV production was significantly increased in Δ*mlaE*Δ*nlpI*.

**Figure 1:**
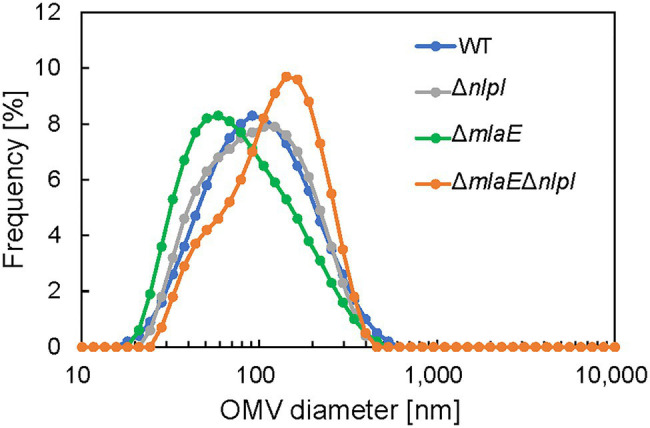
Distribution of outer membrane vesicle (OMV) diameter in each *Escherichia coli* strain. The OMV samples were collected after 24 h in culture. The diameter of the OMVs was determined using dynamic light scattering (DLS).

Next, the diameter of the OMVs in each strain was measured *via* DLS, and the size distribution of the OMVs was plotted (Figure 1). The WT strain had a normal size distribution, with a peak value of approximately 8% at approximately 90 nm; this distribution agreed with previous research (Alves et al., 2016). The average OMV size was 83.9 ± 0.3 nm (Table 2). The values are the means of average values from three independent experiments. The distribution of the OMVs of Δ*nlpI* strain almost overlapped with that of WT strain; the average size of Δ*nlpI* OMVs was 81.7 ± 0.3 nm, comparable with that of the WT strain. Meanwhile, the distribution of the Δ*mlaE* OMVs shifted to a smaller value, with a peak value of 8% at approximately 60 nm. The average OMV size of Δ*mlaE* was 65.9 ± 0.6 nm. These data suggested that Δ*mlaE* produced smaller OMVs. By contrast, the distribution of Δ*mlaE*Δ*nlpI* shifted to a larger value, with the peak value of 10% at approximately 100 nm. Furthermore, the plot of Δ*mlaE*Δ*nlpI* strain was not a normal distribution; it had a shoulder at approximately 70 nm. The average OMV size was 100.4 ± 0.5 nm, significantly larger than that of WT strain. Therefore, the size distributions of the OMVs differ between the knockout mutant strains.

The cell volume of each strain was measured at 3 and 24 h postinoculation using qNano and plotted in histograms (Supplementary Figure S1). At 3 h, the plot of Δ*nlpI* shifted to a smaller value than the others. The cell volume of the WT strain was 1.08 ± 0.27 fL, consistent with that observed in *E. coli* cells, at 1.16 fL, in a previous study (Yamada et al., 2017). The cell volumes of Δ*mlaE* and Δ*mlaE*Δ*nlpI* strains were 1.04 ± 0.27 and 1.08 ± 0.28 fL, respectively. The deletion of these genes likely did not affect the cell volume in the growth phase. By contrast, the cell size of Δ*nlpI* was slightly smaller than the others at 0.85 ± 0.26 fL.

At 24 h, all the cell volume plots also followed a normal distribution; however, the plots of the mutant strains shifted to a smaller value compared with that of the WT strain. The cell volume of WT was 0.52 ± 0.18 fL, smaller than its volume at 3 h, consistent with a previous report that the cell volume of *E. coli* decreased to approximately 0.5 fL in a medium with poor nutrients (Kubitschek, 1990). Thus, it is reasonable that the cell size decreases in the stationary phase. The cell volumes of Δ*nlpI*, Δ*mlaE*, and Δ*mlaE*Δ*nlpI* were 0.39 ± 0.15, 0.41 ± 0.14, and 0.39 ± 0.20 fL, respectively. Therefore, the cell volume was significantly smaller in the mutant strains at the end of the culture.

### Observation of *E. coli* Cells Using Quick-Freeze, Deep-Etch EM

Since the cell size changed in the hypervesiculating strains, there might be a major change in the appearance of the cells. QFDE-EM is a powerful tool for investigating the spatial structure of bacterial envelopment (Tulum et al., 2019); it has been applied to analyze the biogenesis of OMV in *B*. *agrestis* (Takaki et al., 2020). Here, the cell structure of each *E. coli* strain was visualized using QFDE-EM. The cells at 24 h were collected, centrifuged, placed on glass, frozen, fractured, deeply etched, and shadowed with platinum. Then, the platinum replicas were recovered and observed.

**Figure 2:**
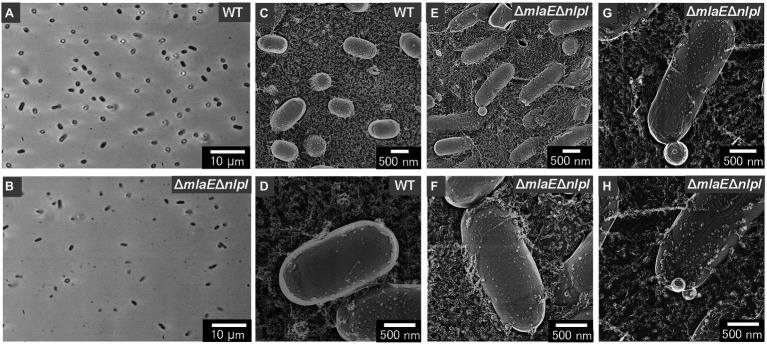
Surface structure of the wild-type (WT) *E. coli* BW25113 and *ΔmlaEΔnlpI* cells visualized *via* quick-freeze, deep-etch electron microscopy (QFDE-EM). **(A,B)** Phase-contrast optical microscopy image of the WT **(A)** and Δ*mlaE*Δ*nlpI* cells **(B)**. **(C)** Field image of the WT cells. **(D)** Magnified image of the surface of WT cells. **(E)** Field image of the *ΔmlaEΔnlpI* cells. **(F–H)** Magnified image of the surface of *ΔmlaEΔnlpI* cells.

The shape and dimensions of the WT cells were consistent with the images of living WT cells obtained by optical microscopy (Figures 2A,C). The surface of the WT cells was observed using QFDE-EM (Figures 2C,D). The Δ*mlaE*Δ*nlpI* cells appeared longer than the WT cells (Figures 2B,E,F); furthermore, several mutant cells were observed to form vesicles (Figure 2E). The magnified images demonstrated that the Δ*mlaE*Δ*nlpI* cells generated OMVs from the tip of their long axis; the diameters of the OMVs were approximately 400 (Figure 2G) and 200 nm (Figure 2H). The size of these OMVs is relatively larger but in the range of the size distribution (Figure 1).

**Figure 3:**
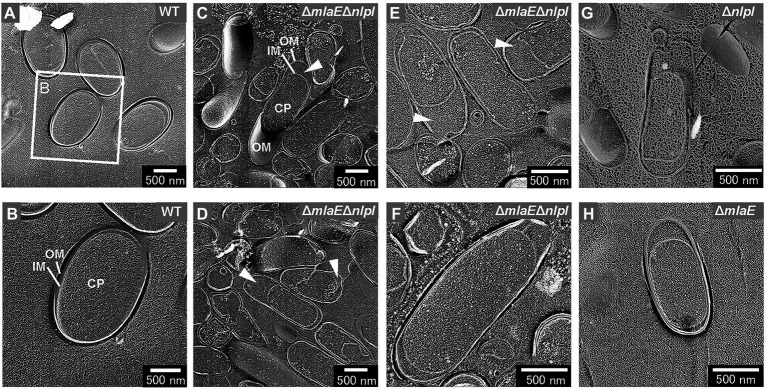
Cross-section of various fractured *E. coli* cells visualized by QFDE-EM. **(A)** Field image of cells. **(B)** Magnified image of the structure of the WT cells. **(C)** Field image of the *ΔmlaEΔnlpI* cells. OM, outer membrane; IM, inner membrane; and CP, cytoplasm. **(D–F)** Magnified image of the structure of the *ΔmlaEΔnlpI* cells. The arrowheads indicate the plasmolysis. **(G)** Magnified image of the structure of the *ΔnlpI* cells. **(H)** Magnified image of the structure of the *ΔmlaE* cell.

The intracellular compartments, i.e., outer membrane, inner membrane, and cytoplasm (CP), of the WT cells were visualized using the freeze-fractured sections without the freeze-etched step (Figures 3A,B). The Δ*mlaE*Δ*nlpI* cells were elongated, and large periplasmic spaces were observed in most cells (Figures 3C,D). The magnified images revealed that plasmolysis was induced in these mutant cells (Figures 3E,F). Plasmolysis is the shrinking of protoplasm away from the cell wall of a plant or bacterium (Woldringh, 1994; Lang et al., 2014). The protoplasmic shrinking is often due to water loss *via* exosmosis, thereby resulting in gaps between the cell wall and the plasma membrane. Clear plasmolysis was observed in approximately 50% or more of the Δ*mlaE*Δ*nlpI* cells. Meanwhile, plasmolysis was also observed in the single-deletion mutants Δ*nlpI* and Δ*mlaE*, although less frequently than in the double-deletion mutant (Figures 3G,H). These results indicate that plasmolysis is a key factor for OMV production.

**Figure 4:**
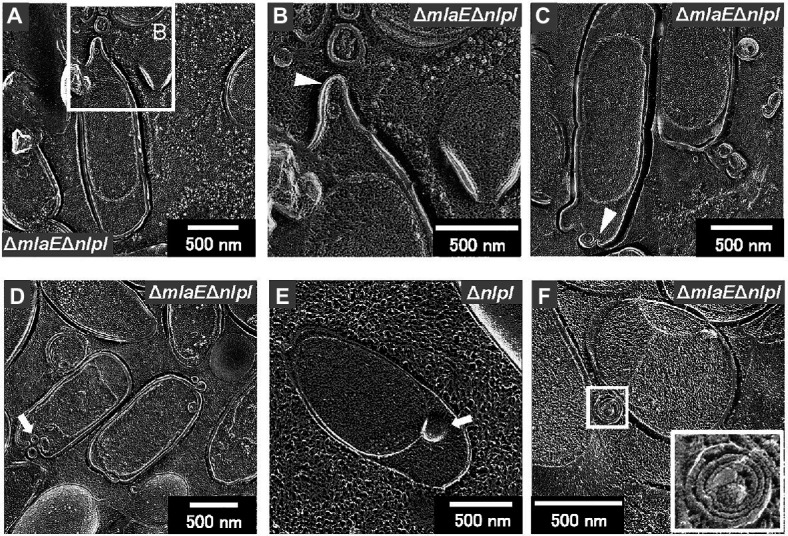
Cross-section of plasmolyzed *E. coli* cells. **(A–D)** Magnified image of the structure of the plasmolyzed *ΔmlaEΔnlpI* cells. The arrowheads indicate the blebbing from the outer membrane. **(E)** Magnified image of the structure of the plasmolyzed *ΔnlpI* cells. The arrows indicate the intracellular vesicle. **(F)** The multilamellar OMV generated from the Δ*mlaEΔnlpI* cells.

**Figure 5:**
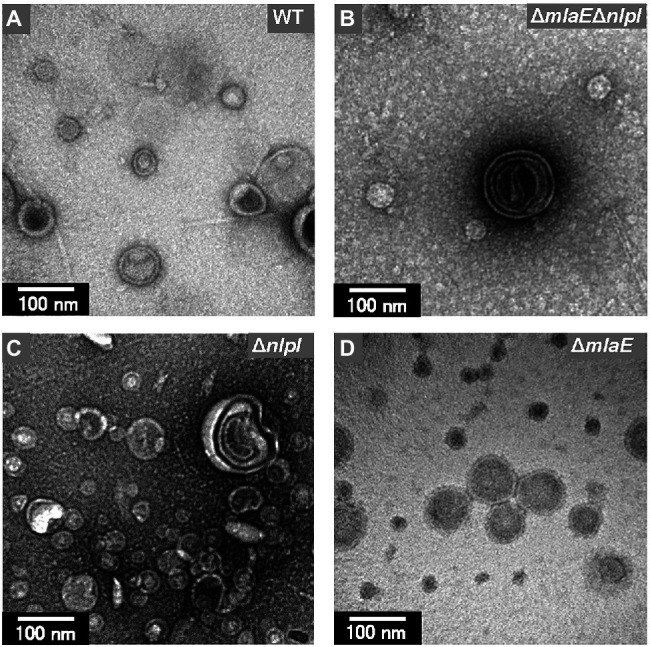
Transmission electron microscopy (TEM) images of the OMVs isolated from *E. coli*. **(A)** WT. **(B)** Δ*mlaEΔnlpI*. **(C)** *ΔnlpI*. **(D)** *ΔmlaE* strains. The OMVs were stained with uranyl acetate.

Further observation revealed that the tip of the plasmolyzed Δ*mlaE*Δ*nlpI* cells was elongated, and OMVs were generated (Figures 4A,B). Since this phenomenon was frequently confirmed in many other cells (Figures 4C,D), it may be the main route of OMV production in the Δ*mlaE*Δ*nlpI* cells. Interestingly, intracellular vesicles in the enlarged periplasmic space were observed in the Δ*mlaE*Δ*nlpI* cells (Figure 4D). In the left cell of Figure 4D; the intracellular vesicle appeared to be produced by the destruction of the inner membrane. By contrast, these intracellular vesicles were observed at an exceptionally low frequency in the Δ*nlpI* cells (Figure 4E), and never observed in the WT and Δ*mlaE* cells. Even more interesting, multilamellar OMVs were observed in the Δ*mlaE*Δ*nlpI* cells (Figure 4F). To further confirm the existence of multilamellar OMVs, the OMVs were isolated by ultracentrifugation and negatively stained with uranyl acetate (Figure 5). The multilamellar OMVs were observed in the Δ*mlaE*Δ*nlpI* sample (Figure 5B). In addition, the multilamellar OMVs were observed in Δ*nlpI* samples at an extremely low frequency (Figure 5C). By contrast, multilamellar OMVs were not observed in the WT and Δ*mlaE* cells (Figures 5A,D). These results are consistent with those from QFDE-EM that intracellular vesicles were observed in the Δ*mlaE*Δ*nlpI* cells, at a low frequency in the Δ*nlpI*, and never observed in the WT and Δ*mlaE* cells.

### Secretion of Intracellularly Expressed GFP Through OMVs

**Figure 6:**
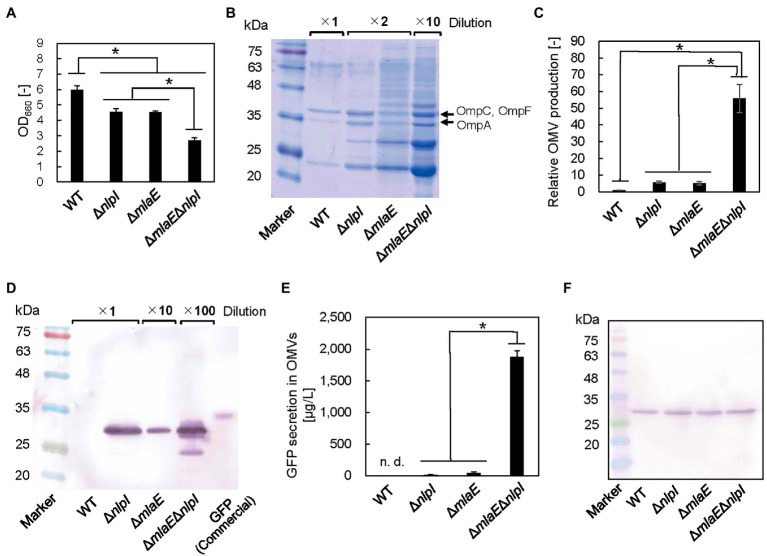
Green fluorescent protein (GFP) secretion in the OMV fraction isolated from each *E. coli* strain. **(A)** OD_660_ of each *E. coli* strain after 24 h in culture. **(B)** Sodium dodecyl sulfate–polyacrylamide gel electrophoresis (SDS-PAGE) analysis of the OMVs isolated from each *E. coli* strain. **(C)** Relative OMV production by each *E. coli* strain normalized to that by the WT strain. **(D)** Western blot analysis of secreted GFP using an anti-His-tag primary antibody. **(E)** Quantitative evaluation of GFP secretion in the OMV fractions of corresponding *E. coli* strains. **(F)** Western blot analysis of the GFP produced in each *E. coli* strain. Loading samples were prepared on an OD_660_ basis to include the same quantity of cells. Values were quantified on the basis of a GFP standard sample. The data are expressed as the mean of three independent experiments (*n* = 3) and SD (vertical bar). An asterisk indicates the statistical significance as determined by ANOVA with Tukey’s test (*p* < 0.05).

To investigate the possibility that the multilamellar OMVs in Δ*mlaE*Δ*nlpI* are OIMVs, the presence of cytosolic components in the multilamellar OMVs was examined by expressing recombinant GFP protein in the cytosol of each *E. coli* strain. In this case, recombinant GFP is not normally incorporated into OMVs because of its lack of a signal peptide. The OD_660_ values of the WT, Δ*nlpI*, Δ*mlaE*, and Δ*mlaE*Δ*nlpI* strains expressing the pCA24N-*gfp* plasmid were measured (Figure 6A). The OD_660_ values of Δ*nlpI* and Δ*mlaE* were 4.6 ± 0.2 and 4.5 ± 0.1, respectively, lower than that of the WT strain at 6.0 ± 0.2. Δ*mlaE*Δ*nlpI* mutant had the lowest OD_660_ at 2.7 ± 0.2.

We also estimated OMV production on the basis of the densitometric analysis of the protein band at approximately 37 kDa that included OmpC, F combined with OmpA (Figures 6B,C). Although the OMV production by Δ*nlpI*/*gfp* and Δ*mlaE*/*gfp* strains was 5.7 ± 0.6 and 5.1 ± 1.0 times, respectively, that of the WT/*gfp* strain, the OMV production by Δ*mlaE*Δ*nlpI*/*gfp* strain was found to be 55.8 ± 8.3 times that of the WT strain. These results suggested that introducing the pCA24N-*gfp* plasmid did not substantially affect the OMV production.

Figure 6D shows the results of western blots of GFP in the OMV fraction. Here, the OMV fraction was first collected as ammonium sulfate precipitate before ultracentrifugation step, which could have also precipitated soluble GFP from the culture supernatants if there were damaged cells and may not necessarily mean that the GFP is located inside the vesicles. However, in the preliminary experiments, the viability for each strain was similar; therefore, the *E. coli* cells were considered to be intact. Additionally, the OMV fraction was washed at ultracentrifugation step. These facts together indicate that the possibility of coprecipitation of soluble GFP is low. As the result, the bands appeared at the expected molecular mass of GFP protein at approximately 27 kDa, except for the WT strain (Figure 6D), suggesting that the OMV fraction from the WT strain did not contain detectable GFP. In the Δ*nlpI*/*gfp* and Δ*mlaE*/*gfp* strains, the same band appeared. This band was much more intense in Δ*mlaE*Δ*nlpI*/*gfp* strain, even after a 1:100 dilution. The amount of GFP secreted through OMVs in the culture medium was quantified on the basis of the densitometry of the band in the western blot against the commercial GFP with a known concentration. Here, the commercial GFP band migrated at approximately 34 kDa. GFP was not detectable in the OMV fraction of the WT/*gfp* strain (Figure 6E). Since the recombinant GFP was produced in the cytosol, it is reasonable that the WT did not secrete GFP *via* OMV. Conversely, GFP secretion by Δ*nlpI* and Δ*mlaE* strains was 14.3 ± 6.8 and 36.5 ± 21.3 μg/L, respectively. The double-deletion mutant Δ*mlaE*Δ*nlpI*/*gfp* secreted GFP at 1.8 ± 0.1 mg/L in the medium, significantly more than other strains. Considering that the secretion of GFP by WT was not detectable and less than that of Δ*nlpI* (14.3 ± 6.8 μg/L), the GFP secretion of Δ*mlaE*Δ*nlpI* is more than 100 times higher than that of WT or Δ*nlpI*, and about 50 times higher than that of Δ*mlaE*. The OMV samples of WT/*gfp* and Δ*mlaE*Δ*nlpI*/*gfp* cultures were observed using CLSM (Supplementary Figure S2). The OMV sample of Δ*mlaE*Δ*nlpI*/*gfp* culture was much more fluorescent, consistent with the results from the western blots.

Because of the extremely high secretion of GFP by the Δ*mlaE*Δ*nlpI*/*gfp* strain, we hypothesized that besides enhancing the OMV production, the deletion of both *mlaE* and *nlpI* genes increased the level of GFP in the *E. coli* cell. The same amount of whole-cell lysates was analyzed in western blotting to compare the GFP production in different cells (Figure 6F). The bands were similarly intense among the strains, suggesting that GFP production per cell unit was comparable. These results indicate that the increased amount of GFP in the OMV fraction of the Δ*mlaE*Δ*nlpI* mutant was due to a promoted secretion of GFP rather than a high expression level in the cells.

### Observation of PGs Using QFDE-EM

The existence of intracellular and multilamellar vesicles in the Δ*mlaE*Δ*nlpI* cells was confirmed. Also, the secretion of an unusual amount of cytosolic GFP into the medium by the Δ*mlaE*Δ*nlpI* cells was verified. These results suggested the generation of a hole in the PG layer might allow the release of the inner membrane with cytosolic components, followed by the formation of intracellular and multilamellar vesicles.

**Figure 7:**
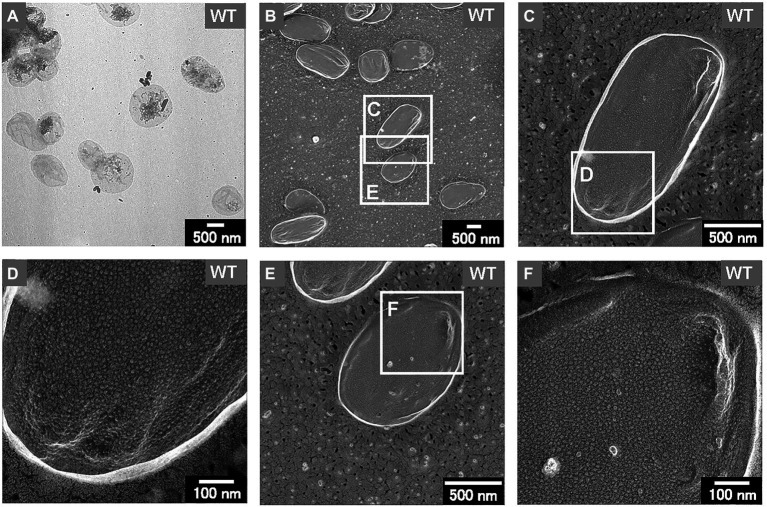
Sacculi of the WT *E. coli* cells. **(A)** The TEM image of the sacculi stained with uranyl acetate. **(B–F)** The surface structures of the sacculi were visualized by QFDE-EM.

Thus, the sacculi of each *E. coli* strain were prepared according to previous research (Vollmer et al., 2008; Jeske et al., 2015). After negative staining with uranyl acetate, the thin sacculi of WT appeared as flat, empty cell envelopes (Figure 7A), as reported previously (Vollmer et al., 2008). The WT sacculi were further observed using QFDE-EM (Figures 7B–F). The surface of the typical WT sacculi appeared remarkably smooth (Figures 7B,C,E). The magnified images confirmed the roughness of the surface of the PG network (Figures 7D,F); however, no obvious holes were observed.

**Figure 8:**
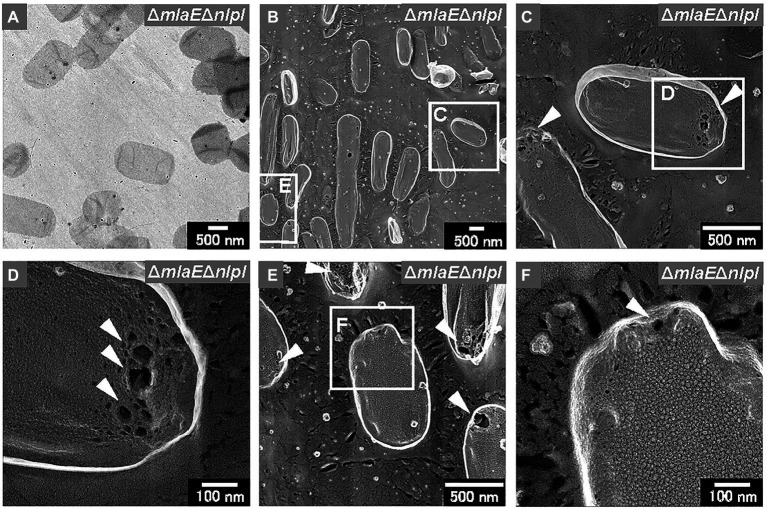
Sacculi of the *ΔmlaEΔnlpI* cells. **(A)** TEM image of sacculi stained with uranyl acetate. **(B–F)** Surface structures of the sacculi were visualized *via* QFDE-EM. The holes on the peptidoglycan (PG) surface are marked by arrowheads.

Sacculi were also successfully prepared from the Δ*mlaE*Δ*nlpI* cells (Figures 8A,B). The sacculi of the double-deletion mutant were longer on the long axis. The magnified QFDE-EM images showed that the Δ*mlaE*Δ*nlpI* sacculi had several holes at the tip of the long axis (Figure 8C). The diameter of the largest hole was more than 50 nm (Figure 8D), substantially larger than the mean radius of the pores for *E. coli* PG, at 2.06 nm, in a previous report (Demchick and Koch, 1996). The holes at the tip of the long axis in the sacculi were confirmed in many other cells (Figure 8E). A magnified image revealed that although the sacculi appeared intact at lower magnifications, they had smaller holes that were noticeably larger than the pores for *E. coli* PG (Figure 8F). Such smaller holes were not observed in the WT sacculi. These results suggested that the generation of the holes in the PG layer of the Δ*mlaE*Δ*nlpI* cells allowed the release of the inner membrane, followed by the intracellular and multilamellar vesicles.

## Discussion

Here, the properties of Δ*mlaE*Δ*nlpI* cells and OMVs were compared with those of the WT and the single-deletion mutants, and an associated mechanism of hypervesiculation and OIMV production was proposed. First, the cell volume of each strain was measured to examine the relationship between OMV production and cell volume. The single and double-gene knockout mutants were shown to have a smaller cell volume at the end of the culture than the WT. However, there was no correlation between cell volume and OMV production in any strain.

Next, the cell structure of the strains was observed. The Δ*mlaE*Δ*nlpI* cells were elongated and vesicles formed from the tip of their long axis. The formation of OMVs from cell tips has rarely been reported. The cross-sections of the fractured cytosol and membrane surface showed that the elongated Δ*mlaE*Δ*nlpI* cells had been plasmolyzed, having large periplasmic spaces. Plasmolysis was also observed in the single-gene knockout mutant Δ*nlpI* and Δ*mlaE* cells, albeit at lower frequency than in the double-gene knockout mutant. Furthermore, intracellular vesicles were frequently observed in the Δ*mlaE*Δ*nlpI* cells, at a low frequency in the Δ*nlpI*, and never observed in the WT and Δ*mlaE* cells.

Thus, this study has demonstrated that plasmolysis and intracellular vesicle production were induced in the hypervesiculating mutant *E. coli* cells. In the case of Gram-negative bacteria, plasmolysis has been observed in the response to moderated hyperosmotic stress with non-permeating solutes (Koch, 1998) and, at least transiently, in the case of higher osmotic shifts with permeating solutes like glycerol (Mille et al., 2002). In the presence of glycerol at approximately 50% (v/v), the *E. coli* cells maintained intact cytoplasmic membrane and produced small intracellular vesicles (Mille et al., 2002). Membrane vesiculation was also observed following severe dehydration involving cell death of *E. coli* (Schwarz and Koch, 1995) and could be facilitated by membrane structural changes like transition from lamellar to non-lamellar phases, combined with the increase in the surface/volume ratio related to water exit (Steponkus, 1999). The characteristics of Δ*mlaE*Δ*nlpI* cells clarified in the study such as plasmolysis and intracellular vesicle production are similar to those when *E. coli* cells are exposed to high osmotic pressure.

Transmission electron microscopy analysis revealed the production of multilamellar OMVs by the Δ*mlaE*Δ*nlpI* cells. These multilamellar OMVs were also confirmed in the Δ*nlpI* cells, albeit at a low level. The multilamellar OMVs may explain why the distribution of OMV size from Δ*mlaE*Δ*nlpI* shifted to a larger value and that the plots did not follow a normal distribution, exhibiting a shoulder at approximately 70 nm.

It is notable that extracellular vesicles produced by Gram-negative bacteria include not only those originating from the outer membrane (OMVs), but also OIMVs and EOMVs, which are defined as OIMVs or EOMVs, respectively (Toyofuku et al., 2019). The first experimental evidence for OIMVs was provided by a transmission electron cryomicroscopy study of *S. vesiculosa* M7 supernatant (Pérez-Cruz et al., 2013) that clearly demonstrated the production of double bilayered OMVs. Meanwhile, the latest study reported that the deletion of *tolB*, encoding part of the Tol-Pal system, led to the production of multiple types of vesicles and increased overall vesicle production in the high vesicle-forming strain *Buttiauxella agrestis* JCM 1090 T (Takaki et al., 2020). The Δ*tolB* mutant strain produces M-OMVs. The visualization of the intracellular compartments of the Δ*tolB* cells *via* QFDE-EM showed that vesicles had accumulated in the large periplasm. The outer membrane formed invaginations to create vesicles, and the precursor of M-OMVs was present in the cell. Hence, multilamellar vesicles may be a characteristic of hypervesiculating strains.

Considering that the deletion of the *nlpI* gene prevented the formation of typical numbers of Lpp-PG crosslinks, there may be holes in the PG of the Δ*mlaE*Δ*nlpI* cells. Through these openings, cytoplasmic membrane material may protrude into the periplasmic space, further releasing these intracellular vesicles into the extracellular space as OIMVs. This hypothesis was tested by expressing recombinant GFP in the cytosol of the Δ*mlaE*Δ*nlpI* cells. As expected, the GFP secretion of Δ*mlaE*Δ*nlpI* is more than 100 times higher than that of WT or Δ*nlpI*, and about 50 times higher than that of Δ*mlaE*, suggesting that cytosolic components were incorporated into OIMVs and discharged into the extracellular space.

**Figure 9:**
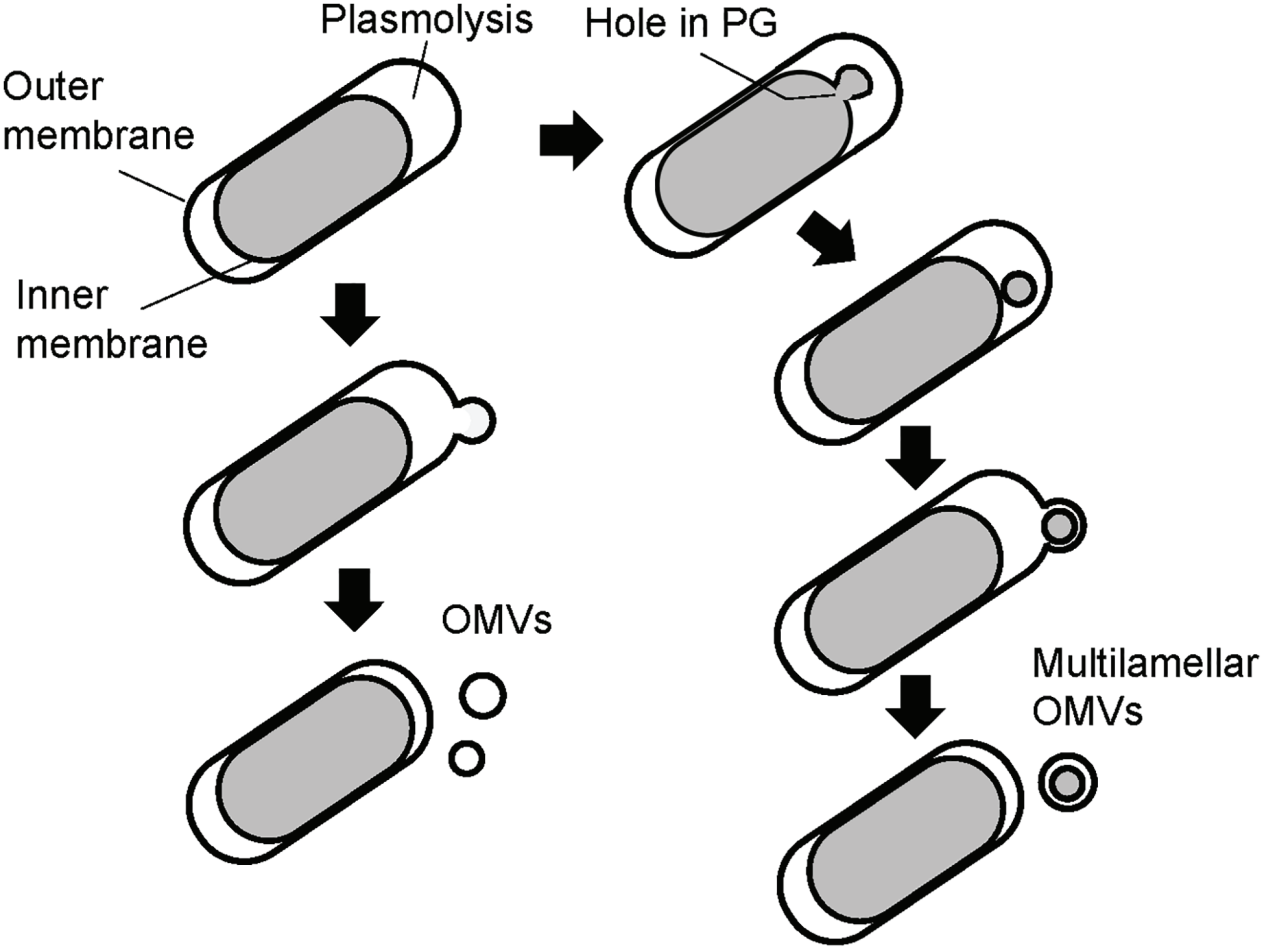
Possible models of the mechanism underlying vesicle formation by Δ*mlaE*Δ*nlpI* cells. Left side: model for mechanism in the promoted unilamellar OMV production. Right side: model for mechanism in OIMV production.

Finally, the PG of the WT and Δ*mlaE*Δ*nlpI* cells was isolated and observed using QFDE-EM. The PG of the WT cells had an exceptionally smooth appearance. By contrast, the surface of a typical Δ*mlaE*Δ*nlpI* PG appeared very wrinkly and contained holes located at the tip of the long axis; a range of diameters of the holes are 10–50 nm. Interestingly, the critical pore size in the Gram-negative PG layer to initiate the bulging of the cytoplasmic membrane has been calculated to be approximately 20 nm (Daly et al., 2011). Some of the holes observed on the Δ*mlaE*Δ*nlpI* PG were larger than 20 nm, indicating that the bulging of the cytoplasmic membrane occurred through large holes and intracellular vesicles was produced. On the basis of these findings, a model of the mechanism underlying the production of OMVs and the release of OIMVs in the Δ*mlaE*Δ*nlpI* strain is proposed (Figure 9). In this model, plasmolysis frequently happens in Δ*mlaE*Δ*nlpI* cells because of the malfunction of Lpp-PG crosslinking. Thus, the extra membrane is removed for producing OMVs. As for the release of the OIMVs, the cytoplasmic membrane material protrudes into the periplasmic space through the PG holes and is released as intracellular vesicles in Δ*mlaE*Δ*nlpI* cells at the first step. Next, the intracellular vesicles are released with the outer membrane, forming multilamellar vesicles. This study provides the microscopic details of the mechanism by which OIMVs are produced by a multigene knockout mutant *E. coli* strain.

## Conclusion

The observation of Δ*mlaE*Δ*nlpI* cells using QFDE-EM revealed that plasmolysis occurred at the tip of the long axis of the cells and that OMVs formed from this tip. Intracellular vesicles and multilamellar OMVs were also observed in the Δ*mlaE*Δ*nlpI* cells. The secretion of recombinant GFP expressed in the cytosol of the Δ*mlaE*Δ*nlpI* cells was more than 100 times higher than that of WT or Δ*nlpI*, and about 50 times higher than that of Δ*mlaE* in the OMV fraction. Additionally, QFDE-EM analysis revealed that the surface of a typical PG layer in the Δ*mlaE*Δ*nlpI* cells had a very wrinkled appearance and many holes. These data suggest that in the Δ*mlaE*Δ*nlpI* cells, cytoplasmic membrane materials protrude into the periplasmic space through the PG holes and are released as intracellular vesicles. Together, the results suggest a mechanism by which the OIMVs produced by Δ*mlaE*Δ*nlpI* cells can include cytosolic components.

## Data Availability Statement

The original contributions presented in the study are included in the article/Supplementary Material; further inquiries can be directed to the corresponding author.

## Author Contributions

YO, MA, and MM proposed the research concept and provided necessary tools for experiments and experimental instructions. YO wrote the manuscript. TS designed and conducted the experiments and analyzed the data. YT and MN conducted the experiments and analyzed the data. All authors contributed to the article and approved the submitted version.

## Conflict of Interest

The authors declare that the research was conducted in the absence of any commercial or financial relationships that could be construed as a potential conflict of interest.

## Publisher’s Note

All claims expressed in this article are solely those of the authors and do not necessarily represent those of their affiliated organizations, or those of the publisher, the editors and the reviewers. Any product that may be evaluated in this article, or claim that may be made by its manufacturer, is not guaranteed or endorsed by the publisher.

## Supplementary Material

Supplementary Figure S1 Distribution of cell volumes in each *E. coli* strain. The cells were harvested after 24 h in culture. Cell volume was determined using qNano.

Supplementary Figure S2 CLSM images of OMV samples isolated from the WT **(A)** and Δ*mlaE*Δ*nlpI* **(B)** strains. The fluorescence levels of OMV in the WT and mutant strains were adjusted to the same range.
