# Supplementary figures and images for: Aberrant Membrane Structures in Hypervesiculating Escherichia coli Strain ΔmlaEΔnlpI Visualized by Electron Microscopy
Source: Front Microbiol. 2021 Aug 11;12:706525. doi: 10.3389/fmicb.2021.706525 (PMC8386018; doi:10.3389/fmicb.2021.706525)

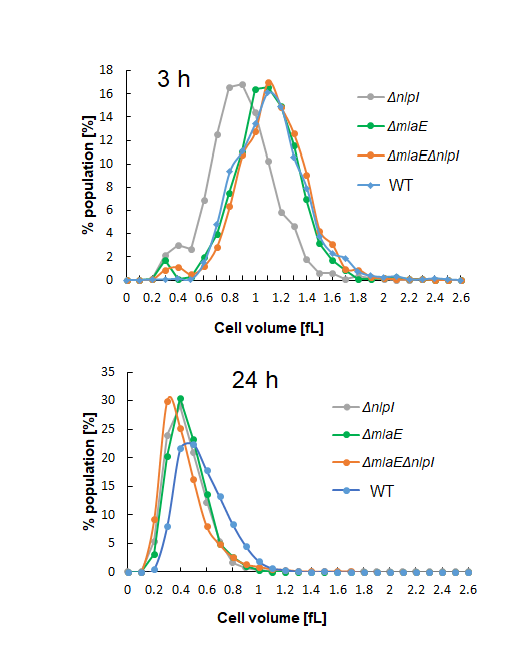

Supplement: Supplementary Figure S1 — Distribution of cell volumes in each E. coli strain. The cells were harvested after 24 h in culture. Cell volume was determined using qNano. [file Image_1.TIF]

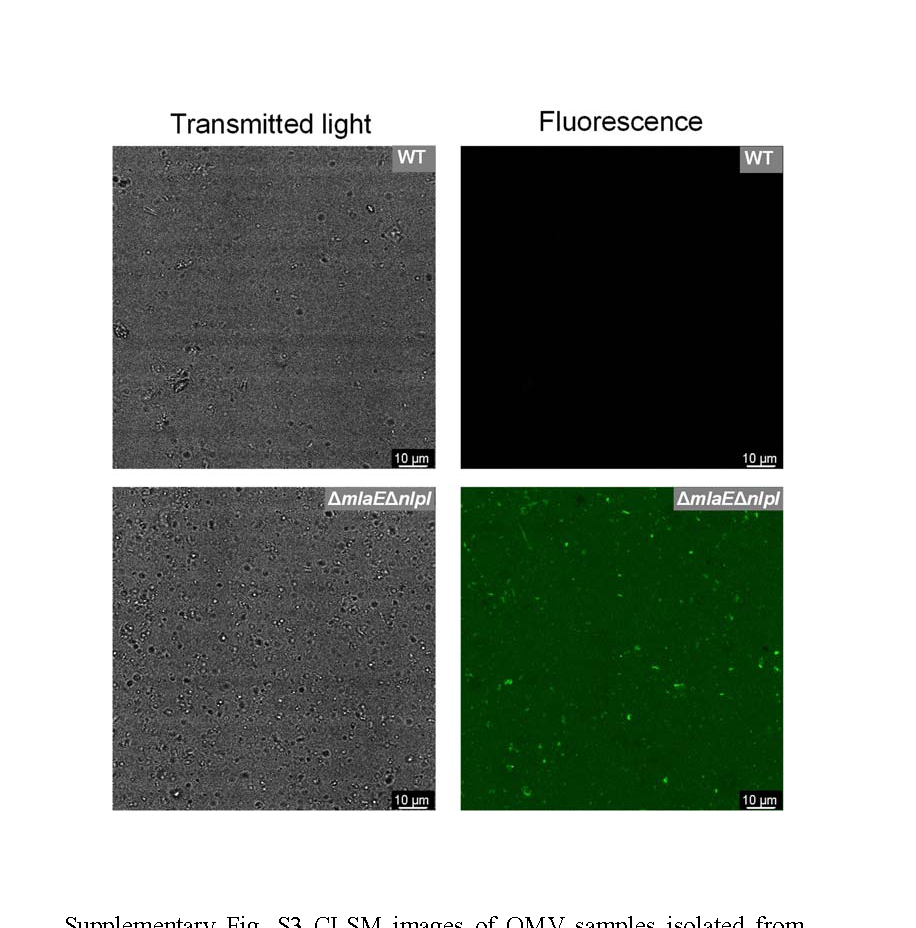

Supplement: Supplementary Figure S2 — CLSM images of OMV samples isolated from the WT (A) and ΔmlaEΔnlpI (B) strains. The fluorescence levels of OMV in the WT and mutant strains were adjusted to the same range. [file Image_2.TIF]
